# Additional considerations for: the impact of frailty on clinical outcomes of older patients undergoing enhanced recovery after lumbar fusion surgery: a prospective cohort study

**DOI:** 10.1097/JS9.0000000000002936

**Published:** 2025-07-02

**Authors:** Aikeremujiang Muheremu, Abudunaibi Aili, Kan Jiang

**Affiliations:** a Orthopedic Research Center, Sixth Affiliated Hospital of Xinjiang Medical University, Urumqi, Xinjiang, China; b Department of Orthopedics, Sixth Affiliated Hospital of Xinjiang Medical University, Urumqi, Xinjiang, China

*Dear Editor,*

We read with great interest the prospective cohort study by Wang *et al*^[1]^ investigating the impact of frailty on postoperative outcomes in older patients undergoing enhanced recovery after surgery (ERAS) for lumbar fusion. The study provides valuable insights into the challenges of managing frail patients in spine surgery and highlights the importance of preoperative frailty assessment. The authors have thoroughly acknowledged several limitations of their work, including the single-center design, lack of a conventional care control group, use of a single frailty assessment tool, and restriction to transforaminal lumbar interbody fusion procedures. While these points are well addressed, we would like to highlight additional considerations that could further strengthen the interpretation and clinical applicability of the findings.

First, while the study adjusted for several clinical factors, additional variables that are not mentioned in this study such as socioeconomic status, caregiver support, and preoperative rehabilitation engagement may influence recovery trajectories in frail patients^[2]^. Future studies could incorporate these factors into multivariate analyses to better isolate the independent effects of frailty.

Second, while the study provides valuable data on 90-day complications and length of stay, longer-term follow-up as long as one postoperative year would help determine whether the benefits of ERAS are sustained in frail patients. Metrics such as mobility, independence in activities of daily living (ADLs), and quality of life (SF-36 or EQ-5D) would complement the existing pain and disability measures.

Third, the ERAS protocol included prehabilitation, but its specific contributions were not analyzed separately. A subgroup analysis of prehabilitation adherence or intensity could identify modifiable targets to optimize outcomes in frail patients^[3]^.

Fourth, the study included patients undergoing 1–5 level fusions, which introduces variability in surgical stress and recovery expectations. Comparing outcomes based on fusion levels could reveal if frailty has a greater effect on recovery from extensive procedures^[4]^.

Besides, two additional considerations also merit attention: while the study met minimum sample size requirements, its statistical power may be insufficient to reliably detect differences in less common but clinically important outcomes, such as major complications, or to support detailed subgroup analyses stratified by frailty severity. By excluding emergency cases and patients with severe comorbidities, the findings may not fully capture frailty’s impact in real-world clinical practice^[5]^. Future studies with broader inclusion criteria and higher patient number could yield findings more applicable to high-risk surgical populations.

In sum, the study by Wang et al. provided important evidence on the challenges of managing frailty in ERAS spine surgery. Addressing these additional considerations in future research—particularly through longer follow-up, broader outcome measures, and more detailed analyses—could refine ERAS protocols and improve care for high-risk older adults.

## Data Availability

Not applicable.
